# Reduced temperature solid-state synthesis of barium sulfide: a greener alternative

**DOI:** 10.1039/d5ra07445b

**Published:** 2025-12-16

**Authors:** William D. Tetlow, Oliver S. Hutter, Marc K. Etherington, Giulia Longo

**Affiliations:** a School of Engineering, Physics and Mathematics, Northumbria University Ellison Place Newcastle upon Tyne NE1 8ST UK marc.k.etherington@northumbria.ac.uk; b Instituto de Diseño y Fabricación, Universitat Politécnica de Valencia (UPV) Camí de Vera, s/n Valencia 46022 Spain glongo@idf.upv.es

## Abstract

Barium sulfide (BaS) serves as a commonly used precursor for advanced barium-based materials, including the emerging perovskite photovoltaic absorber BaZrS_3_. However, conventional BaS production methods are highly energy-intensive, requiring temperatures well exceeding 1000 °C and emitting large quantities of CO_2_ and SO_2_, and raising valid environmental concerns. This work presents a novel solid-state synthesis route for BaS that drastically reduces the environmental and energy demands. By employing a finely milled mixture of barium hydroxide [Ba(OH)_2_] and elemental sulfur, we achieve an efficient conversion (90%) to BaS at a remarkably low annealing temperature of 500 °C. The process is enabled by a low-pressure annealing environment, which facilitates the rapid vaporisation of H_2_O byproducts while maintaining a controlled sulfur partial pressure. This prevents unwanted side reactions and enhances conversion efficiency by continuously removing waste gases, including water vapour and trace amounts of SO_2_, while preserving optimal reaction conditions. The success of the process was confirmed through X-ray diffraction, and aided by Fourier transform infrared spectroscopy, and Raman spectroscopy. The extent of conversion was quantitatively determined using Rietveld refinement of the diffraction patterns. This method offers a more sustainable and economically viable pathway for BaS production. Residual gas analysis has shown a significant reduction in CO_2_ and SO_2_ production compared to earlier processes.

## Abstract 2

Low-toxicity synthesis of BaS at temperatures 500 °C lower than other solid-state reactions.

## Graphical abstract

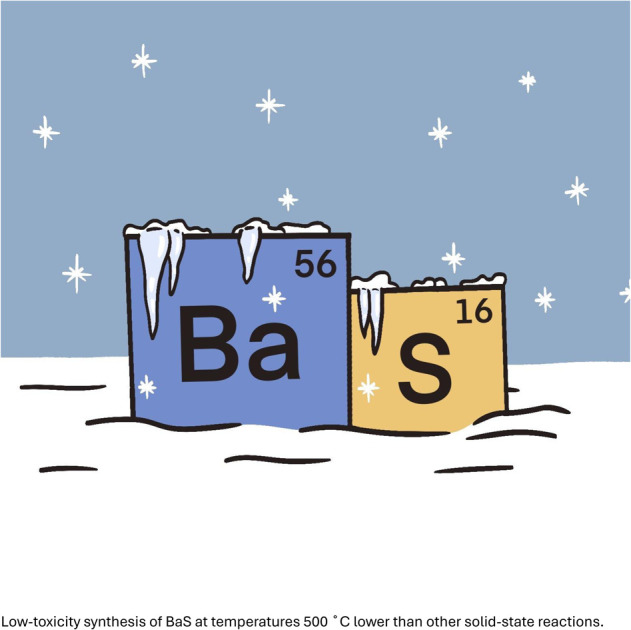

## Introduction

Barium sulfide (BaS) holds importance in various industrial and research applications, ranging from wastewater treatment^1^ to phosphor manufacturing^2^ and synthesis of materials such as BaTaS_3_.^3^ The useful properties of BaS, such as its ability to efficiently remove sulfates from solution^1^ and its uses as a precursor for promising photovoltaic materials^4^ – such as barium-based perovskites BaZrS_3_ and BaSnS_3_,^5–9^ shows its relevance and importance in industry and research.

Among the above-mentioned perovskites, BaZrS_3_, a highly promising chalcogenide perovskite with strong potential for photovoltaic absorber materials, has been synthesised using BaS as precursor.^5,6^ Regardless of the status of the end product, whether in powder or thin film form, the development and understanding of BaS for BaZrS_3_ has the potential for huge impact on the low-temperature synthesis of BaZrS_3_ devices. It has been shown that controlling the BaS_*x*_ polymorphs formation created through the BaS–S reaction during the perovskite synthesis, can sensibly reduce the conversion temperatures.^8–10^ Despite its utility, the synthesis of BaS has historically been constrained by the high temperatures and polluting waste products typically required in conventional methods, with temperatures often exceeding 1100 °C^11^ and producing large quantities of CO_2_.^12^

One of the more common methods of synthesising this material in industry is *via* a carbothermal reduction with coke or another carbon source (eqn (1)), again at temperatures typically exceeding 1100 °C.^11^

1 BaSO_4_ + 2C → BaS + 2CO_2_

Mulopo *et al.* showed this process and its application to the treatment of sulfate-rich sludge from mines.^12^ Optimal process conditions were mapped out, with an ideal molar ratio of C : BaSO_4_ of 2.8 : 1, and temperature of 1028 °C. This process is run for 35 minutes, and the BaS yield was found to be significant at 78.5%. However, these processes require substantial amounts of energy, which is too often generated from unsustainable sources like fossil fuels.

To avoid excess CO_2_ production, sulfur can be used in place of the carbon source, producing SO_2_ as product (eqn (2)).

2 BaSO_4_ + 2S → BaS + 2SO_2_

Zhang *et al.* successfully demonstrated the conversion of BaSO_4_ to BaS through sulfurisation,^13^ as evidenced by distinct XRD peaks of BaS. At 1127 °C, the BaSO_4_ characteristic peaks persisted alongside emerging BaS peaks, resulting in a conversion rate of 61%. Subsequent heating to 1227 °C led to a notable decrease in BaSO_4_ and a corresponding increase in BaS relative peak intensity, with reported conversion reaching 92%.

With the aim of reducing reaction temperature, a more reactive sulfur source, H_2_S, can be effective for BaS synthesis at temperatures below even 200 °C. Tang *et al.* demonstrated successful synthesis *via* bubbling H_2_S gas through a hexane solution containing Ba[N(*t*-Bu)(TMS)]_2_(THF)_2_ as a barium source, for 1 hour.^14^ After successful reaction and the precipitation of BaS, the powder was then held at ∼200 °C for several hours in inert atmosphere to decompose any remaining unwanted compounds. The lower temperature reaction is possible due to the low ionization potential of H_2_S, leading to a highly reactive gas that is quick to dissociate,^15^ even at very low temperatures (∼80 °C). Removing the need to cleave sulfur atoms from allotropes reduces the necessary energy for reaction. However, for most applications, H_2_S is not appropriate for repeated use. Unfortunately, the low temperature of this process comes at a cost as H_2_S has been proven to be highly toxic,^16^ as well as extremely corrosive leading to slow equipment damage.^17^ The work is solution-based and therefore does not push the low temperature boundaries of a fully solid-state BaS synthetic method.

In this work we address the high energy consumption and environmental impact of traditional solid-state BaS synthesis by developing a novel approach. By leveraging mechanochemical activation^18,19^ we combine barium hydroxide and elemental sulfur using ball milling, followed by thermal treatment in a low-pressure environment. This method significantly reduces the required conversion temperature, offering a more energy-efficient and environmentally friendly alternative to conventional processes.^12,13^

Our findings demonstrate the effectiveness of this innovative approach in producing high-quality BaS powder with remarkable efficiency – achieving 90% conversion at 500 °C – while drastically reducing the production of harmful waste gases, such as CO_2_ and SO_2_. This novel method not only lowers energy consumption but also minimizes the environmental impact, positioning it as a promising solution for more sustainable industrial practices. The results highlight the potential of this process for future applications in both industry and research.

## Experimental

### Materials

Barium hydroxide octahydrate Ba(OH)_2_·8H_2_O (CAS: 12230-71-6) was purchased from Sigma Aldrich and used as received. According to the supplier specifications, the material contained up to 0.8 wt% strontium as an impurity and ≤2 wt% barium carbonate (BaCO_3_). Other heavy metal contaminants were present at concentrations below 0.1 wt%. Sulfur powder (CAS: 7704-34-9) was obtained from Sigma Aldrich with a stated purity of 99.98% (trace metal basis). The materials were used without further purification.

### Powder preparation

**Fig. 1:**
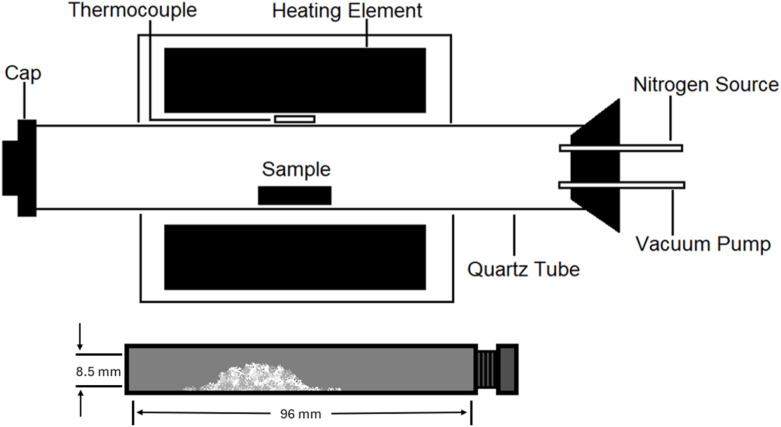
Schematic diagram of tube furnace for sample annealing and graphite vial that holds sample powders.

The desired amount of Ba(OH)_2_·8H_2_O and S were weighed and inserted into a planetary ball-mill where they were ground for different times according to experimental parameters. Approximately 100 mg of ball-milled reaction powder was loaded into custom designed graphite tubes produced at Mersen UK. These graphite tubes were 100 mm in length with outer diameter 13 mm and inner diameter 8.5 mm, see Fig. 1. The tubes were closed at one end and had a threaded opening at the other end, for a graphite screw cap which could be adjusted to allow different size openings. Unless specified, the lid was screwed on the minimum amount as to not fall off at any stage of the process and equated to an 8 mm gap.

### Ball milling

The precursor materials (S and Ba(OH)_2_·8H_2_O) were weighed in the desired ratio and ground with a pestle and mortar to better start the mixing process and discourage unmixed clumps forming in the mill. 1.5–3.0 g of the mixed powder was loaded in a ZrO_2_ jar together with six zirconia balls of 1 cm diameter and then transferred to a planetary ball mill. This was run at 600 rpm, for different amounts of time. During milling, kinetic and thermal energy transfer caused partial dehydration of barium hydroxide hydrate, leading to some moisture release that caused powder clumping. To maintain a homogeneous mixture, checks were performed at 30-minutes intervals to break up clumps and prevent material from sticking to the crucible walls.

### Annealing

After ball milling, the resulting fine precursor powder was transferred into a partially closed graphite tube (as shown below in Fig. 1), which was then annealed in a Carbolite GHC 12/450 horizontal tube furnace (Carbolite Gero, UK). This single-zone furnace has a 450 mm heated length and a maximum temperature of 1200 °C. Annealing was conducted under vacuum with a controlled ramp rate of 10 °C min^−1^. The loaded graphite vial was moved to the centre of the tube furnace, positioned between the heating elements (Fig. 1). The furnace was sealed, and the vacuum pump reduced pressure within the furnace to approximately 1.0 × 10^−3^ mbar before heating began. When using a hydrate such as Ba(OH)_2_·8H_2_O the pumping took significantly longer due to the water content subliming before reaching target pressure. The initial heating stage ramped up at a rate of 10 °C per minute, taking 50 minutes to reach the maximum temperature of 500 °C (773 K), followed by the desired dwell time, holding a constant peak temperature. After the furnace had been given substantial time to cool down to room temperature, the chamber was vented with nitrogen and the sample was retrieved and characterized without further purification or modification.

### Gas emission analysis

To verify the gases produced during the reaction we connected an inductively coupled plasma optical emission spectroscopy (ICP-OES) system (Gencoa Optix) to the exhaust of the system to monitor the species produced during the process. This monitoring was in real-time (sampling every 5 seconds) across the full process from the start of the heating ramp to the dwell and for a duration of the cooling cycle (when significant changes stopped). This was only performed on the completely optimised reaction conditions of 500 °C dwell temperature, 60 minutes dwell time, 2 hour ball milling and 2 : 3 Ba(OH)_2_ : S ratio. Two control analyses were also performed on a sulfur only containing tube and an empty tube (blank). For calculation of the molar fraction emissions for H_2_O, CO_2_ and SO_2_, the partial pressures of each species were integrated by area over the full time of the measurement. The values were then normalised to the total of the three species (with the assumption that other species are just atmospheric contributions or impurities that do not contribute to the reaction).

### X-ray diffraction and analysis

X-ray powder diffraction (XRD) analysis was conducted using a Rigaku SmartLab instrument equipped with Cu Kα radiation (*λ* = 1.5418 Å) to assess the crystalline phases present in the samples. Phase identification and quantification were performed using HighScore Plus software *via* Rietveld refinement.^20,21^ The analysis was used to determine the phase composition of samples after thermal treatment, enabling estimation of conversion efficiency. Refinement included all crystalline phases expected from precursors and products, with the background modelled using a polynomial function and peak shapes fitted using pseudo-Voigt profiles. Scale factors obtained from the refinement were used to calculate the relative weight percentages of each phase. These values were then used to estimate the extent of conversion from precursor to product (mol%). The refinement figures of merit are reported in Table S1, with Table S2 and Fig. S1 representing the approach to estimating the variance values for the refinement. The refinements and the corresponding residuals are presented in Fig. S2–S10. The reference .cif files used to perform the refinement were obtained from ICSD and Materials Project database, as specified in Fig. S11.

### Raman analysis

Raman spectroscopy was performed using a HORIBA LabRAM Soleil confocal Raman microscope with a green laser excitation source (532 nm) and laser power of 1.5 mW with the use of 1.6% ND filter. Spectra were collected at room temperature with a 5× objective lens, 5 second acquisition time and 5 accumulations. A silicon film was used as reference to calibrate and account for any peak shifts.

### FTIR analysis

Fourier-transform infrared (FTIR) spectra were collected in attenuated total reflectance (ATR) mode using a Bruker Alpha spectrometer.

## Results and discussion

The proposed reaction for the BaS synthesis that is presented in this work is as follows:

3 Ba(OH)_2_·8H_2_O → Ba(OH)_2_ + 8H_2_O^↑^ ∼250 °C

4 2Ba(OH)_2_ + 3S → 2BaO + 3S + 2H_2_O^↑^ ≤ 410 °C^22^

5 2BaO + 3S → 2BaS + SO^↑^_2_ > 410 °C^22^

This reaction relies heavily upon a unique low-pressure furnace environment and custom designed graphite tube (see Fig. 1). When the graphite tube is filled with sulfur and the screw cap is loosely closed, it simultaneously allows for the creation of sulfur pressure inside the chamber whilst providing a sufficient gap for pressure to escape to avoid tube explosions. The continuous vacuum within the furnace also ensures removal of the gas product from the reaction environment, favouring the product formation through Le Chatelier's principle.^23^

Based on the above principles we hypothesise that the hydrate barium hydroxide is completely dehydrated at around 250 °C.^22^ The exact temperatures of Ba(OH)_2_·8H_2_O dehydration and sulfur sublimation within the sample vial is not known. However, it has been investigated that Ba(OH)_2_·8H_2_O dehydration begins at temperatures as low as 30 °C (under dynamic vacuum environment)^22^ leaving behind only the Ba(OH)_2_ and S mixture (with sulfur that starts subliming at 67 °C).^24^

Under inert (typically 1 atm of nitrogen or argon), atmospheric conditions, barium hydroxide decomposition requires temperatures of approximately 410 °C^22^ and leads to the formation of BaO + H_2_O.

BaO is known to be stable in only very dry conditions and will rapidly react with surrounding moisture to re-form Ba(OH)_2_.^25^ However, given the low-pressure in the furnace and continually running vacuum pump, the H_2_O released from the hydrated hydroxide will be removed as water vapour^26^ before the hydroxide decomposition temperature is reached, making reformation of Ba(OH)_2_ difficult. After this stage, the BaO is left under remaining sulfur partial pressure in the dry tube before hitting the peak temperature of the reaction. This leads to a favourable reaction between BaO and S, with the formation of BaS.

In the next section we discuss the optimisation of four key parameters that we identified as significant for the synthesis of BaS *via* this method.

### Optimisation strategy

To obtain the best conversion for the lowest temperature possible *via* this solid-state method we decided to optimise four key parameters:

• Annealing temperature (300–500 °C).

• Dwell time (5–60 minutes).

• Ba(OH)_2_ : S molar ratio (1 : 3 to 2 : 3).

• Ball milling duration (0–6 h).

**Fig. 2:**
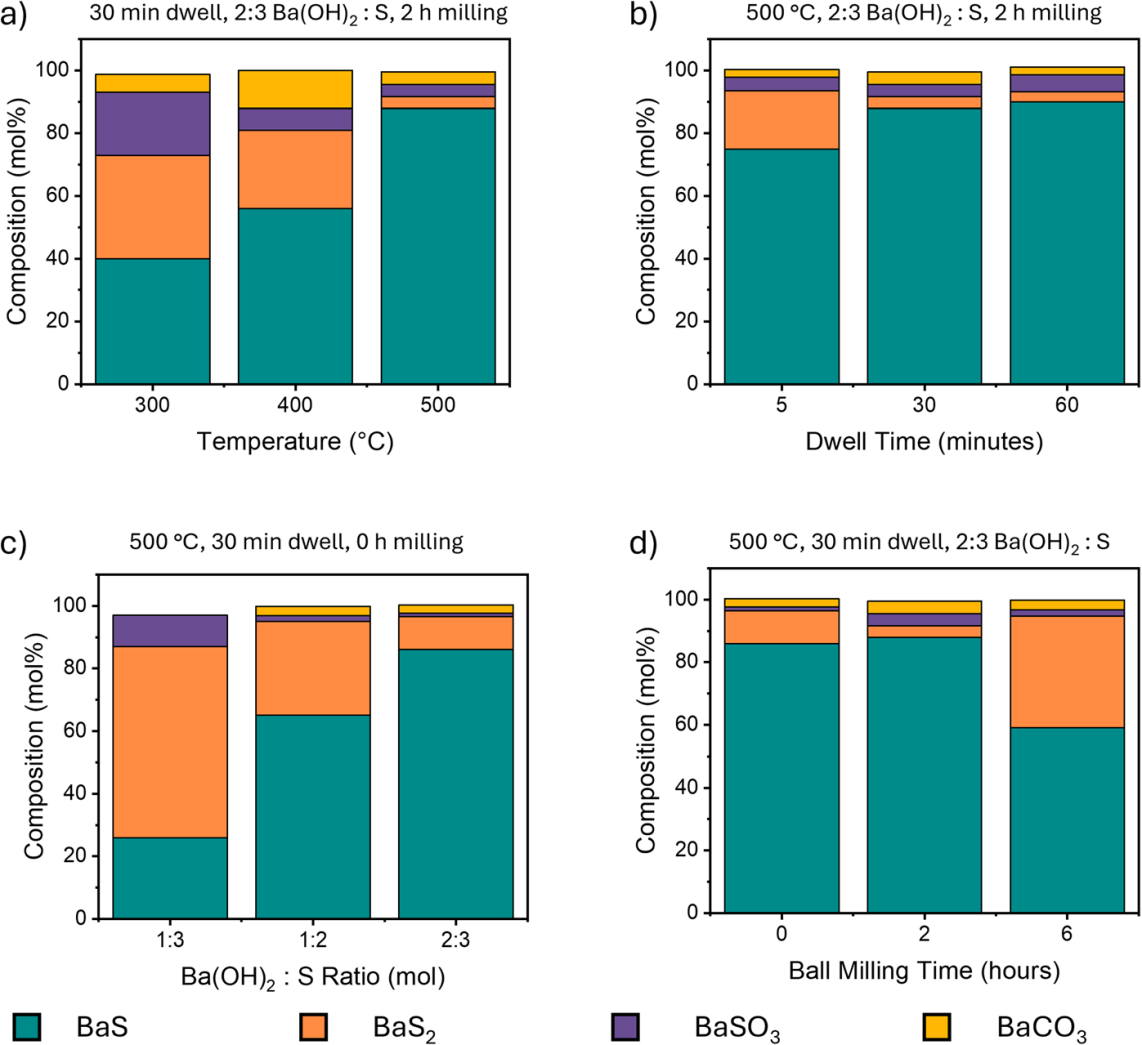
Systematic optimisation of BaS synthesis conditions. Stacked bar charts showing phase composition (mol%) as a function of (a) annealing temperature, (b) dwell time at 500 °C, (c) Ba(OH)_2_ : S ratio and (d) ball milling duration. Optimal conditions (500 °C, 30–60 minutes, 2 : 3 Ba(OH)_2_ : S ratio and 2 hours milling) yield 88–90% BaS with minimal impurities.

An overview of the results from these studies as a function of the key parameters are shown in Fig. 2. The products were characterised through multi-technique validation that combined XRD, Raman and FTIR and the overall optimal conditions were identified as 500 °C with a dwell time of between 30 and 60 minutes at a stoichiometric Ba(OH)_2_ : S ratio of 2 : 3 with 2 hours of ball milling. The following sections examine each parameter in more detail followed by mechanistic insights and an initial assessment of the exhaust gases produced by this reaction pathway.

### Temperature variation

**Fig. 3:**
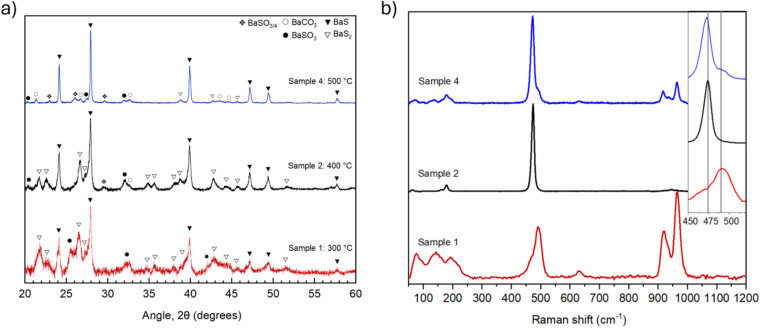
Temperature-dependent phase evolution during BaS synthesis. (a) XRD diffractograms of barium hydroxide-sulfur mixtures (2 : 3 Ba(OH)_2_ : S ratio) annealed for 30 minutes at: 500 °C (Sample 4, blue), 400 °C (Sample 2, black), 300 °C (Sample 1, red). Difference in signal-to-noise ratio between samples is a consequence of annealing duration and crystallinity of final product. (b) Corresponding Raman spectra showing evolution from BaSO_3_ dominated (300 °C, peaks at 967 and 917 cm^−1^) to BaS_2_ intermediate (400 °C, peak at 474 cm^−1^) to BaS dominated (500 °C, Raman-inactive). Inset: detail of peak shift indicating phase transition.

Since the aim of this work is to develop a greener approach to BaS synthesis, we first investigated the temperature limits necessary to achieve sufficient conversion. Given that the decomposition temperature of barium hydroxide is known to be 410 °C, we sought to push the boundaries of this reaction. Fig. 3 presents the XRD patterns for samples annealed at peak temperatures of 300, 400, and 500 °C, illustrating the impact of increasing temperature on phase purity and crystallinity.

At the lower limit, Sample 1 (Table 1 and Fig. 3a) was annealed at 300 °C for 30 minutes. The diffractogram exhibits high background noise and low overall peak intensity, indicating poor crystallinity. Although key BaS peaks are present, conversion is limited to 40%, with a significant fraction (33%) of BaS_2_ remaining, suggesting that BaS_2_ is a stepping stone in the formation of BaS. The formation of BaS, which should not happen at this temperature in an equilibrium reaction,^27^ is likely favoured by the dynamic environment of our reaction chamber, as previously discussed. Unreacted barium hydroxide is not readily observed, but the low sample crystallinity makes the identification and refinement of low concentration species challenging.

**Table 1:** Table of conversion results for samples 1–5, where annealing temperature is varied between 300–500 °C and the peak dwell time is varied between 5–60 minutes. Sulfur ratio and all milling parameters were kept constant: 2 : 3 (Ba(OH)_2_: S (moles)), 2 h milling at 600 rpm

Sample ID	Annealing temperature (°C)	Time (minutes)	BaS conversion (mol%)	Remaining phases (mol%)
1	300	30	40% ± 1%	BaS_2_: 33% ± 1%, BaSO_3_: 20% ± 1%, BaCO_3_: 5.7% ± 0.5%
2	400	30	56% ± 2%	BaS_2_: 25% ± 1%, BaSO_3_: 7% ± 1%, BaCO_3_: 12% ± 1%
3	500	05	75% ± 1%	BaS_2_: 18.5% ± 0.2%, BaSO_3_: 4.4% ± 0.2%, BaCO_3_: 2.4% ± 0.1%
4	500	30	88% ± 1%	BaS_2_: 3.7% ± 0.2%, BaSO_3_: 3.8% ± 0.3%, BaCO_3_: 4.0% ± 0.1%
5	500	60	90% ± 1%	BaS_2_: 3.2% ± 0.2%, BaSO_3_: 5.4% ± 0.3%, BaCO_3_: 2.5% ± 0.1%

At 400 °C, Sample 2 (Table 1 and Fig. 3a), the diffractogram shows a clear enhancement in peak intensity and signal-to-noise ratio. The increased crystallinity allows for improved identification of individual phases, with BaS conversion reaching 56%. Residual BaS_2_ is still present at 25%, similar to BaSO_3_ traces, but the relative intensity of BaS peaks has improved, suggesting a more complete reaction compared to Sample 1.

The most significant improvement is observed in Sample 4 (Table 1 and Fig. 3a), annealed at 500 °C. A five time increase in the BaS peaks counts compared to Sample 2 demonstrates a dramatic improvement in phase purity. BaS dominates the diffraction pattern, and all characteristic BaS_2_ peaks (see Fig. S11 for reference) disappear, confirming full conversion. The pattern features sharp peaks and minimal background noise, allowing for precise phase identification. At this temperature, BaS yield reaches 88%, marking a substantial improvement over lower-temperature reactions.

These observations are supported by the Raman spectra reported in Fig. 3b. BaS is not Raman active, but the byproducts identified by XRD (BaS_2_, BaSO_3_, BaCO_3_ and BaSO_4_) are. The Raman spectrum of Sample 1 shows the characteristic peaks of BaSO_3_, with the asymmetric and symmetric stretching at 967 and 918 cm^−1^, and the *ν*_2_ and *ν*_4_ bending modes located at 630 and 490 cm^−1^.^28,29^ The peak at 490 cm^−1^ presents a shoulder at lower chemical shift, at around 465 cm^−1^, due to inversion-induced splitting.^30^ The peaks below 200 cm^−1^ derive from the Ba–O vibrations.^31^ The presence of BaSO_3_ rather than BaSO_4_ is confirmed by the absence of peaks above 1000 cm^−1^ (Fig. 3b) and is further supported by FTIR spectrum of Sample 1 (Fig. S12), which shows the characteristic bands of BaSO_3_ as reported in literature.^32^ Additional small peaks in the FTIR are assigned to the impurities present in the chemicals used in this work (BaCO_3_).

As the synthetic temperature is increased in Sample 2, the Raman spectrum shows a drastic intensity reduction of the BaSO_3_ peaks, with the appearance of an intense peak located at 474 cm^−1^, with no evidence of splitting. We assign this peak to BaS_2_ A_g_^4^ vibration mode, calculated to be at 450 cm^−1^.^6^ The difference between the Sample 1 and 2 peak location can be appreciated from the inset in Fig. 3b. The residual presence of some BaSO_3_ is supported by the FTIR (Fig. S12), which additionally shows a reduction of the signals coming from the previously mentioned impurities.

The Raman spectrum of Sample 4 shows a decrease in the relative intensity of the most intense peak located at 475 cm^−1^, due to a decrease of BaS_2_ in favour of BaS, which is not Raman active. Similarly, the BaSO_3_ peaks at 917 cm^−1^ and 967 cm^−1^ are more evident than in Sample 2 not due to an increased concentration of this species, but rather to the reduction of BaS_2_ signal and the consequent shadowing of the peaks at higher and lower chemical shift.

Conclusions from spectroscopic analysis are therefore well aligned with the XRD analysis, supporting the quantifications extracted by refinement. Moreover, these results underscore the necessity of high-temperature processing for efficient BaS formation, establishing 500 °C as a critical threshold for optimising conversion and crystallinity in this greener synthetic route.

### Time variation

**Fig. 4:**
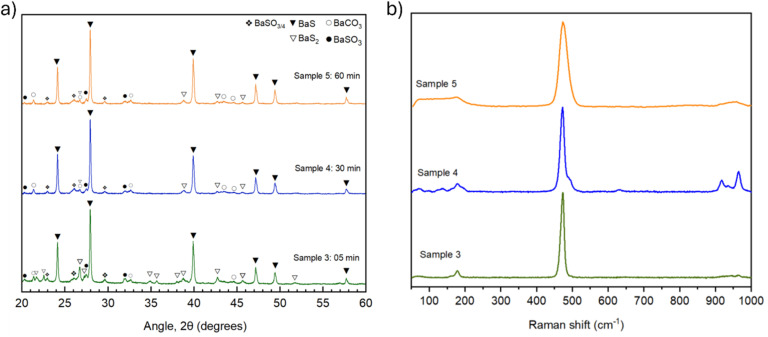
Time-dependent conversion kinetics at 500 °C. (a) XRD diffractograms of precursor powder (2 : 3 Ba(OH)_2_ : S ratio) annealed at: 500 °C for 60 minutes (Sample 5, orange), 30 minutes (Sample 4, blue), and 05 minutes (Sample 3, green). (b) Corresponding Raman spectra demonstrating continuous decrease in BaS_2_ signal intensity (474 cm^−1^) with extended dwell time, while BaS (Raman-inactive) increases.

We then investigated the impact of peak temperature hold time on BaS synthesis, specifically focusing on the dwell times of 5, 30, and 60 minutes at 500 °C. The results are presented in Fig. 4.

The results clearly demonstrate that the conversion process continues to improve with increasing dwell time, but with diminishing returns. Sample 3 (Table 1 and Fig. 4a), held at 500 °C for only 5 minutes, achieved 75% BaS conversion, with a significant proportion (18.5%) of the product remaining as BaS_2_. As the dwell time increased to 30 minutes, Sample 4 (Table 1 and Fig. 4a), conversion improved to 88%, and the quantity of BaS_2_ was markedly reduced. The greatest conversion, 90%, was achieved with a 60-minutes hold time, Sample 5 (Table 1 and Fig. 4a), where BaS_2_ was nearly absent. This would suggest that BaS_2_ is favourably formed at the beginning of the reaction and later converted as the high temperature dwell time is longer. Similarly to before, this is further supported by the Raman and FTIR spectra reported in Fig. 4b and S13.^6^ Additionally, the relative intensity between the BaS_2_ and BaO peaks in the Raman, presented in Table S3, reveals a continuous reduction of the BaS_2_ signal, leading to compositional estimations that lay very close to the ones obtained through Rietveld refinement. These results are in good agreement with previous studies which show that between 300 and 400 °C, and moderate sulfur pressure, BaS_2_ is the thermodynamically favourable species, decomposing to BaS as temperature increases.^27^

The slow ramp-up rate (approximately 10 °C min^−1^) and even slower cooling rate of the furnace (approximately −3 °C min^−1^) meant that the impact of dwell time on conversion was amplified, as the furnace's cooling rate limited the ability to control rapid temperature shifts. As the dwell time increased, the sample spent more time within the temperature range where BaS conversion was most efficient. While a 60-minutes dwell time resulted in the highest conversion observed, for the purposes of this study, a 30-minutes dwell time at 500 °C was selected as the benchmark for evaluating sulfur ratios and related parameters. This choice provides a consistent basis for comparison while still achieving high conversion (88% BaS) with minimal BaS_2_. Further optimisation of dwell time may yield incremental improvements in purity, and the trade-off between processing time and phase conversion is left open for future consideration.

### Sulfur ratio

**Fig. 5:**
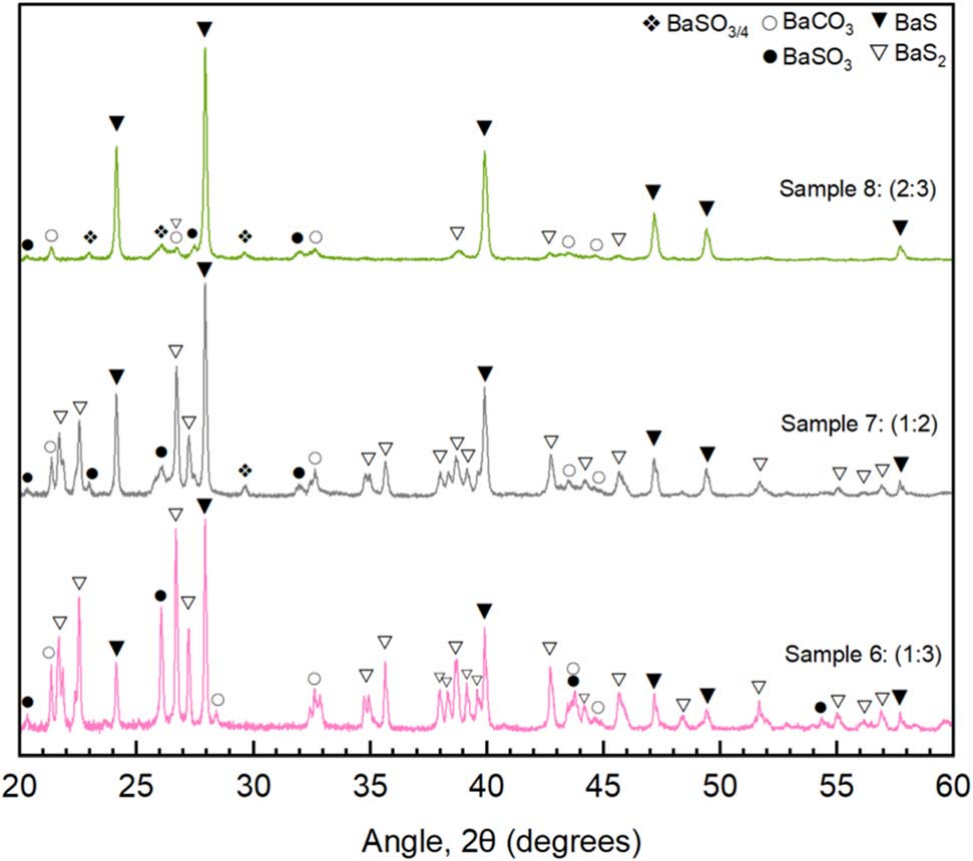
XRD diffractograms of annealed samples (500 °C for 30 minutes) with varying quantities of sulfur: 2 mol Ba(OH)_2_ per 3 mol sulfur (Sample 8, light green), 1 mol Ba(OH)_2_ per 2 mol sulfur (Sample 7, grey), 1 mol Ba(OH)_2_ per 3 mol sulfur (Sample 6, pink).

Corrado *et al.* demonstrates the formation of BaS_*x*_ polymorphs is highly sensitive to sulfur partial pressure,^27^ making sulfur ratios an important variable to consider. Fig. 5 shows three samples (6, 7, 8) of different Ba(OH)_2_ : S ratios (1 : 3, 1 : 2, 2 : 3) to demonstrate the impact on conversion. For all samples the annealing temperature and dwell time were kept at 500 °C and 30 minutes respectively.

Given that this experimental setup allows for the escape of some sulfur gas, a larger than stoichiometric sulfur ratio was expected to be required for full conversion. Starting with Sample 6, 3 moles of S for every mole of Ba(OH)_2_ was trialed, which resulted in 61% BaS_2_ formation and only 26% BaS conversion (Sample 6, Table 2 and Fig. 5). As predicted through quantum chemical simulations,^27,33^ BaS_3_ formation would require more than 10^3^ Pa (10 mbar) of sulfur vapour pressure at these temperatures (500 °C), yet no BaS_3_ is formed, despite the favourable stoichiometry. This leads us to the assumption that the sulfur vapour pressure within the vessel does not exceed 10^3^ Pa (10 mbar). As the sulfur ratio is brought down – closer to stoichiometric – the desired phase, BaS, becomes more prevalent, increasing to 65% conversion at a 1 : 2 Ba(OH)_2_ : S ratio in Sample 7 (Table 2 and Fig. 5). A reduction of sulfur to a 2 : 3 ratio proves most effective, leading to 86% conversion for Sample 8 (Table 2 and Fig. 5). These results are in good agreement with computational studies, which predicted the favourable formation of BaS_2_ over BaS for higher sulfur pressures and also suggest that appreciable sulfur pressure can be built in our graphite tubes with screwed lid.^27^

**Table 2:** Table of conversion results for Samples 6–10, where annealing temperature (500 °C) and dwell time (30 minutes) were kept constant. Milling duration was varied between 0 hours (which was only hand mixed in a pestle and mortar for 2 minutes) and 6 hours of milling at 600 rpm. The sulfur ratio added to the hydroxide was varied between 1 : 3, 1 : 2 and 2 : 3 (Ba(OH)_2_:S)

Sample #	BM duration (hours)	S Ratio (Ba(OH)_2_:S) (mol)	BaS conversion (mol%)	Remaining phases (mol%)
6	0 h	1 : 3	26% ± 1%	BaS_2_: 61% ± 1%, BaSO_3_: 10% ± 1%
7	0 h	1 : 2	65% ± 1%	BaS_2_: 30% ± 1%, BaSO_3_: 1.8% ± 0.4%, BaCO_3_: 3.1% ± 0.2%
8	0 h	2 : 3	86% ± 1%	BaS_2_: 10.5% ± 0.2%, BaSO_3_: 1.2% ± 0.2%, BaCO_3_: 2.6% ± 0.1%
4	2 h	2 : 3	88% ± 1%	BaS_2_: 3.7% ± 0.2%, BaSO_3_: 3.8% ± 0.3%, BaCO_3_: 4.0% ± 0.1%
9	6 h	2 : 3	59% ± 1%	BaS_2_: 35.8% ± 0.3%, BaSO_3_: 1.9% ± 0.1%, BaCO_3_: 3.2% ± 0.1%

### Ball milling time

**Fig. 6:**
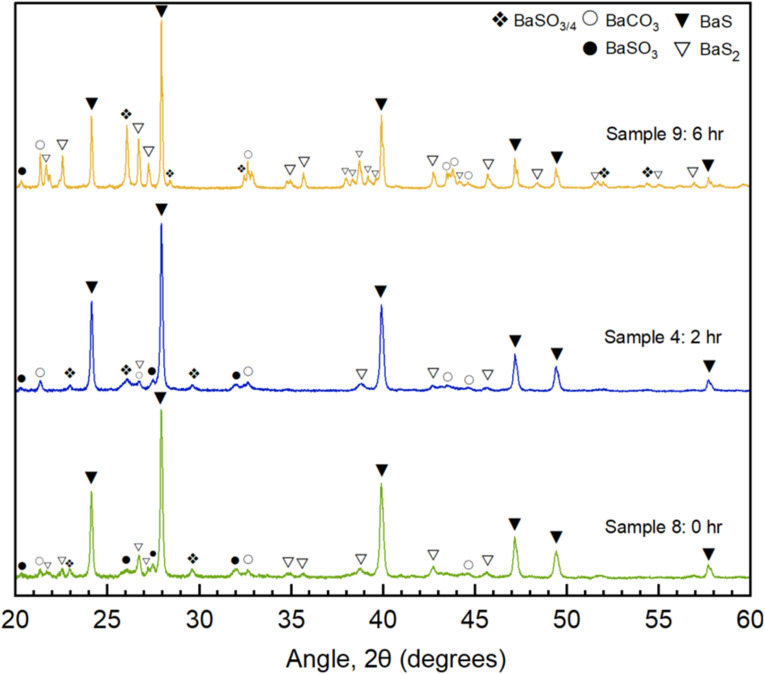
XRD diffractograms of precursor powder (2 : 3 Ba(OH)_2_ : S ratio) annealed at 500 °C for 30 minutes after milling at 600 rpm for different durations: 6 hours milling (Sample 9, yellow), 2 hours milling (Sample 4, blue), 0 hours milling (Sample 8, light green).

To further refine this approach, we investigated the effect of ball milling duration on precursor activation. The results are reported in Fig. 6.

Ball milling introduces mechanochemical activation, which can enhance reaction kinetics by increasing surface area, reducing particle size, and promoting intimate mixing of reactants. However, as shown in Table 2 and Fig. 6, there exists a ‘Goldilocks zone’ for milling duration—where too little or too much milling adversely affects conversion.

Sample 8 (Table 2 and Fig. 6), which was not milled before annealing, still exhibited a strong diffraction pattern, achieving 86% conversion at 500 °C for 30 minutes. This suggests that thermal processing alone can drive a substantial degree of conversion. Sample 4 (Table 2 and Fig. 6), subjected to 2 hours of milling, demonstrated a modest improvement, increasing BaS conversion to 88%. However, the diffraction pattern showed sharper peaks and reduced background noise, indicative of enhanced crystallinity.

Interestingly, over-milling negatively impacted conversion, as seen in Sample 9 (Table 2 and Fig. 6). With an extended milling duration, BaS conversion dropped to 59%, accompanied by a substantial increase in BaS_2_ formation (35.8%). The corresponding diffractogram revealed intensified BaS_2_ peaks (see Fig. S11 for reference), suggesting an alternative reaction pathway was becoming more dominant.

The presence of oxygen during the ball milling (conducted in air) favours oxidation instead of sulfurization, as evidenced by the increased sulfate peaks, reducing the yield of BaS. Additionally, the decrease in conversion may be attributed to excessive reduction in sulfur particle size, leading to a lower energy threshold for sulfur vaporization. As milling duration increases, rapid vaporization of sulfur occurs at a lower temperature, resulting in a mismatch between peak sulfur vapour pressure and the intended reaction dwell time. This phenomenon is similar to what was observed in Sample 3 (Table 1 and Fig. 4), which was only held at 500 °C for 5 minutes and demonstrated similarly poor conversion. Furthermore, prolonged milling introduces risks such as moisture release from hydroxide hydrates, which can promote clumping, and potential carbonate formation due to prolonged exposure to atmospheric CO_2_.

These results highlight the critical error of over-milling in BaS synthesis, demonstrating that an optimal duration exists to maximize conversion efficiency. While moderate milling improves conversion and phase purity, excessive milling disrupts reaction kinetics and reduces yield. Thus, maintaining milling within the optimal range is essential for achieving high-purity BaS in a scalable, greener synthesis approach.

### Importance of controlled gas flow

A critical aspect of this work is the graphite tube cap configuration we implemented to make use of Le Chatelier's principle. Table 3 shows the impact on BaS conversion of identically prepared samples just by partially opening the tube. Both samples have the same precursor composition of 2 : 3 Ba(OH)_2_ : S, a milling time of 2 hours with annealing temperature and time of 500 °C and 30 minutes respectively. The only variable is that Sample 10 is a closed system with the cap fully tightened and Sample 4 has a loose fit and is the standard protocol for this work.

**Table 3:** Table showing the impact of a fully tightened lid (Sample 10) *vs.* the loose fit used throughout the process (Sample 4)

Sample #	Temp (°C)	Time (minutes)	BaS conversion (mol%)	Remaining phases (mol%)
10 – Fully tightened lid	500	30	32% ± 1%	BaS_2_: 25% ± 1%, BaSO_3_: 27.1% ± 0.5%, BaCO_3_: 16.4% ± 0.3%
4 – Loose fit	500	30	88% ± 1%	BaS_2_: 3.7% ± 0.2%, BaSO_3_: 3.8% ± 0.3%, BaCO_3_: 4.0% ± 0.1%

It is immediately apparent the sheer scale of the impact with BaS conversion for the closed system being at 32%, a value that rises to 88% in the loose fit system (Sample 4). This is an almost 3-fold increase in BaS yield simply from cap configuration and is visible in the difference in quality of the XRD diffractograms (see Fig. S14).

The fully tightened lid (Sample 10) traps the gasses released during synthesis with the primary issue being the water vapour accumulation from Ba(OH)_2_·8H_2_O dehydration (eqn (3)). The consequence of the trapped H_2_O is that it prevents Ba(OH)_2_ decomposition meaning that eqn (4) is suppressed and rehydration, the reverse of eqn (4), is promoted. There is thus an unfavourable reaction equilibrium due to product accumulation and Le Chatelier's principle works against the desired reaction.

The loose fit approach combines two critical design ideas:

(1) Maintains sulfur pressure inside the tube (prevents excessive sulfur loss).

(2) Allows gas to escape to vacuum (removes H_2_O and SO_2_).

This loose fit approach allowed controlled monitoring of the gas exhaust species which is explored in the following section.

### Real-time emission monitoring and residual gas analysis (RGA)

**Fig. 7:**
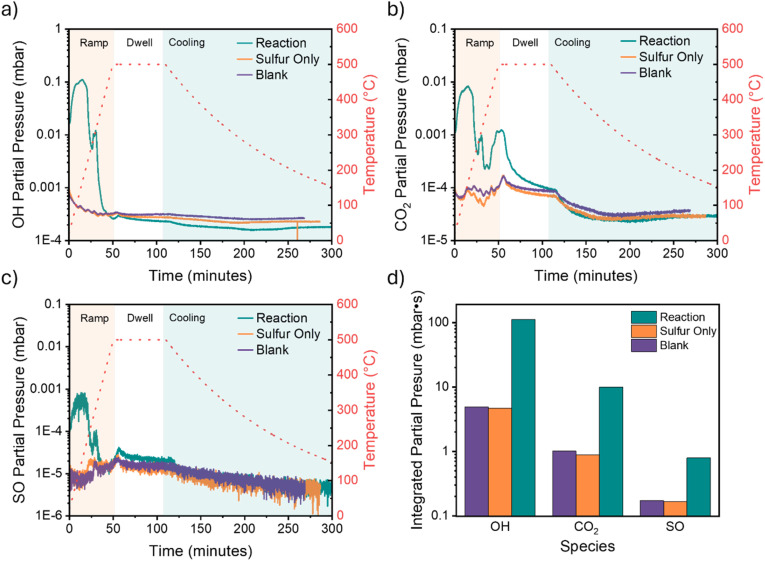
The partial pressures measured during the furnace run of a reaction filled tube, sulfur only tube and empty tube (blank) monitoring (a) OH, (b) CO_2_ and (c) SO. The integrated partial pressures over the full run are shown in (d).

To directly quantify the environmental impact and validate the reaction mechanism we utilised a Gencoa Optix residual gas analyser that utilises optical emission spectroscopy to monitor elements and species of exhaust systems. By splitting off a channel of the furnace exhaust we were able to monitor the species produced by the reaction throughout the whole process. After pumping down the quartz tube to the correct pressure the plasma measurement was activated in time with the timed furnace procedure so that the exhaust species could be mapped to the reaction both as a function of time and temperature. This monitoring was performed on Sample 5 (reaction), as it had the largest overall conversion of BaS, and two controls: a tube with sulfur only (sulfur only); and an empty tube (blank). The results from these measurements are shown in Fig. 7. The key species of OH, CO_2_ and SO have been highlighted in these plots to demonstrate the impact of the reaction conditions on the exhaust gases. As is clear from the three plots Fig. 7a–c and the summary bar charts (Fig. 7d), these three species are heightened during the reaction and thus are chemically related to the process. The OH and SO peaks are roughly consistent with the expected reaction, with the elevated SO pressure during the dwell time being consistent with the reaction persisting beyond the removal of all the water vapour. The large pressures of all three species during the ramp process are likely a result of outgassing and the beginning of the reaction according to eqn (3). The CO_2_ is most likely due to reaction between the water vapour and the graphite crucible and is again a good indication of the reaction taking place.^34,35^ A full breakdown of all the gases present in the reaction run are shown in Fig. S15. The wavelengths of the peaks monitored for the respective species are shown in Table S4.

Compared to conventional BaS synthesis methods, our approach demonstrates substantial environmental benefits. The carbothermal reduction process presented by Mulopo *et al.*^12^ produces mainly CO_2_ (4 mol CO_2_ per mol BaS), while sulfurisation methods such as in Zhang *et al.*^13^ generates mainly SO_2_ (2 mol SO_2_ per mol BaS). In contrast, our Ba(OH)_2_-based synthesis produces primarily water vapour. Real-time residual gas analysis (Fig. 7) demonstrated that volatile emissions comprise approximately 91% H_2_O, 8.1% CO_2_, and 0.65% SO_2_ in molar fractions.

## Conclusion

This study demonstrates an optimised method for synthesising barium sulfide (BaS) with conversion efficiencies approaching 90%, while significantly reducing reaction temperatures by approximately 50–60% (∼400 °C) compared to conventional solid-state synthesis methods. This reduction not only enhances energy efficiency but also minimises environmental impact by reducing CO_2_ and SO_2_ emissions typically associated with BaS production. Through real-time RGA of the exhaust we have identified that the process primarily produces water vapour due to the use of Ba(OH)_2_ as a reactant, with some CO_2_ and SO_2_ produced but in a much-reduced amount.

Through systematic variation of reaction parameters, we identified 500 °C with a 30 minutes dwell time as the optimal condition for high-purity BaS synthesis. While increasing dwell time to 60 minutes may further improve conversion, the marginal gain must be balanced against energy efficiency considerations. The role of ball milling was also explored, revealing that moderate milling (2 hours) enhances conversion, but excessive milling (*e.g.*, 6+ hours) has the opposite effect, reducing overall yield. These findings establish a clear processing window for efficient BaS formation.

Beyond BaS synthesis, the study also highlights the potential for controlled formation of BaS_2_ under specific conditions. Given the theoretical interest in BaS_3_ as a precursor for BaZrS_3_, further exploration of BaS_2_ and BaS_3_ synthesis could provide new insights for perovskite material development. Future work should focus on refining these reaction pathways while maintaining the energy and environmental advantages demonstrated in this study.

## Conflicts of interest

There are no conflicts to declare.

## Supplementary Material

RA-015-D5RA07445B-s001

## Data Availability

Data for this article are available at figshare.northumbria.ac.uk at https://doi.org/10.25398/rd.northumbria.29281655. Supplementary information (SI): further XRD refinement, reference diffractograms, FTIR, Raman analysis and residual gas analysis. See DOI: https://doi.org/10.1039/d5ra07445b.
