# Supplementary material for: Reduced temperature solid-state synthesis of barium sulfide: a greener alternative
Source: RSC Adv. 2025 Dec 16;15(58):50446–56. doi: 10.1039/d5ra07445b (PMC12706637; doi:10.1039/d5ra07445b)
Supplement: RA-015-D5RA07445B-s001 [file RA-015-D5RA07445B-s001.pdf]

Supplementary Information (SI) for RSC Advances.
This journal is © The Royal Society of Chemistry 2025

Supplementary Information

Reduced Temperature Solid-State Synthesis of Barium Sulfide: A Greener Alternative

William D. Tetlow[1], Oliver S. Hutter[1], Marc K. Etherington[1*], Giulia Longo[1,2*]

[1]School of Engineering, Physics and Mathematics, Northumbria University, Ellison Place, Newcastle upon Tyne, NE1 8ST, UK.
[2] Instituto de Diseño y Fabricación, Universitat Politécnica de Valencia (UPV), Camí de Vera, s/n 46022, Valencia, Spain.

Contents

REFINEMENT 3
REFERENCE DIFFRACTOGRAMS 14
FTIR 15
RAMAN ANALYSIS 16
RESIDUAL GAS ANALYSIS 17
REFERENCES...19

REFINEMENT

Table S1: *R_{wp}, R_{exp} and Chi-squared (χ^2) values for each refined sample.*

Sample ID	R_{wp}	R_{exp}	χ^2
1	0.22060	0.12372	1.7830
2	0.31077	0.16097	1.9306
3	0.27752	0.09465	2.9321
4	0.25919	0.09712	2.6688
5	0.25234	0.10331	2.4426
6	0.35485	0.22033	1.6105
7	0.32152	0.19210	2.1677
8	0.29191	0.08516	3.0676
9	0.36116	0.08349	4.3257

The parameters refined in the refinement process are as follows:

- Instrument broadening (peak shape parameters U, V, W, calibrated using LaB_6 standard)
- Background (Chebyshev polynomial function)
- Zero shift
- Scale factor
- Lattice parameters (a, b, c)

The refinement via HighScore Plus attributes standard deviation error values for each phase for each sample at around 0.1%. However, this error does not reflect the variance between samples. For this, we performed multiple XRD measurements and subsequent refinements on the same sample. The XRD patterns and the corresponding calculated compositions are reported in Figure S1 and Table S2.

Table S2: *Variance values for each phase via a sample (2:3 $Ba(OH)_2$: S ratio), ball milled for 2 hours and annealed at 500 ˚C for 30 minutes.*

Sample ID	BaS (mol%)	BaS_2 (mol%)	$BaSO_3$ (mol%)	$BaCO_3$ (mol%)
A	85.2	3.30	8.10	3.40
B	86.2	4.66	6.50	2.64
C	90.0	4.3	2.9	2.9
Variance	4.8	1.36	5.2	0.76

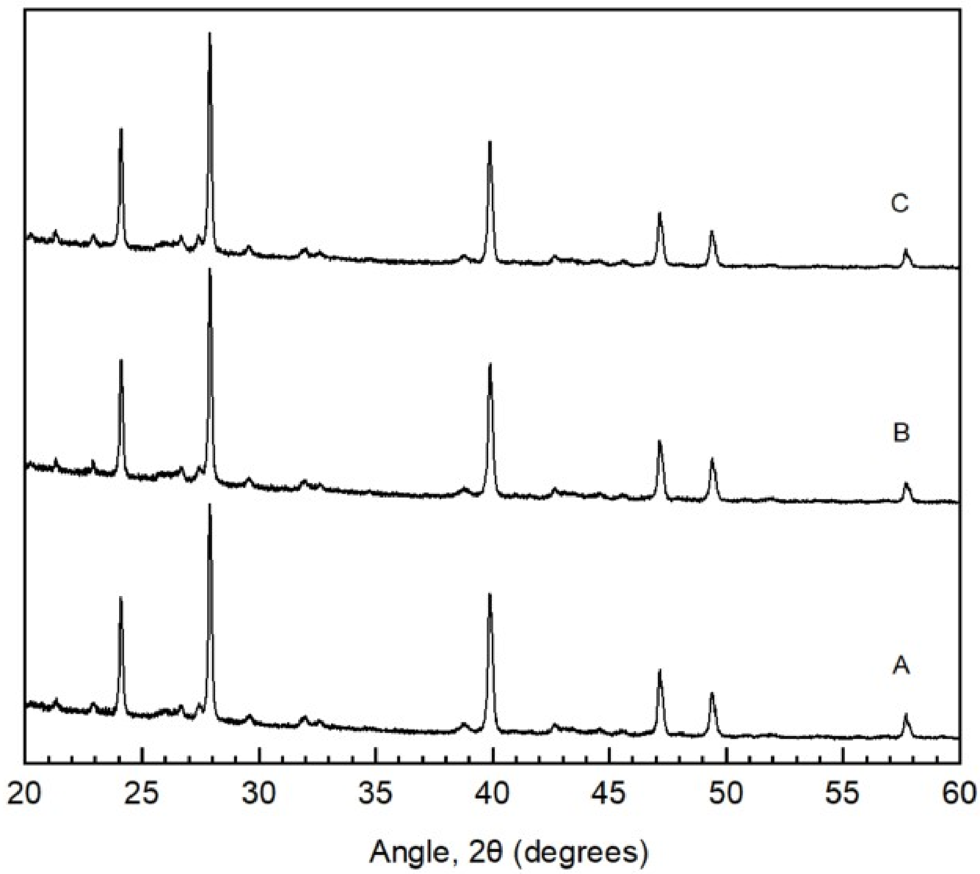

***Figure S1**: XRD diffractograms of the same powder measured multiple times (samples A, B and C), used for variance calculations in Table S2.*

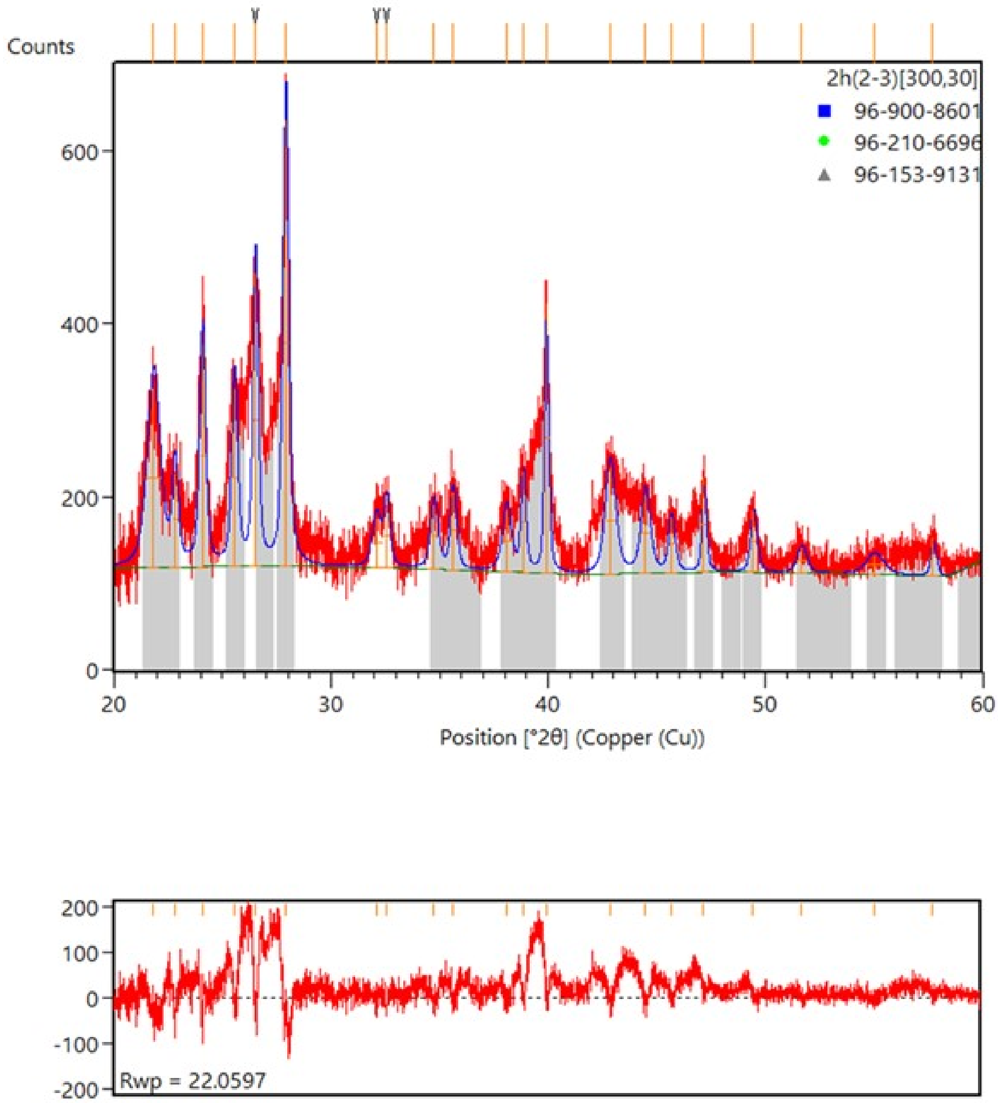

Figure S2: *Top: Experimental (red) and calculated (blue) XRD diffractograms of Sample 1: [300 °C, 30 mins](2:3* $Ba(OH)_2$ *: S ratio, 2h milling). Bottom: Refinement residual plot.*

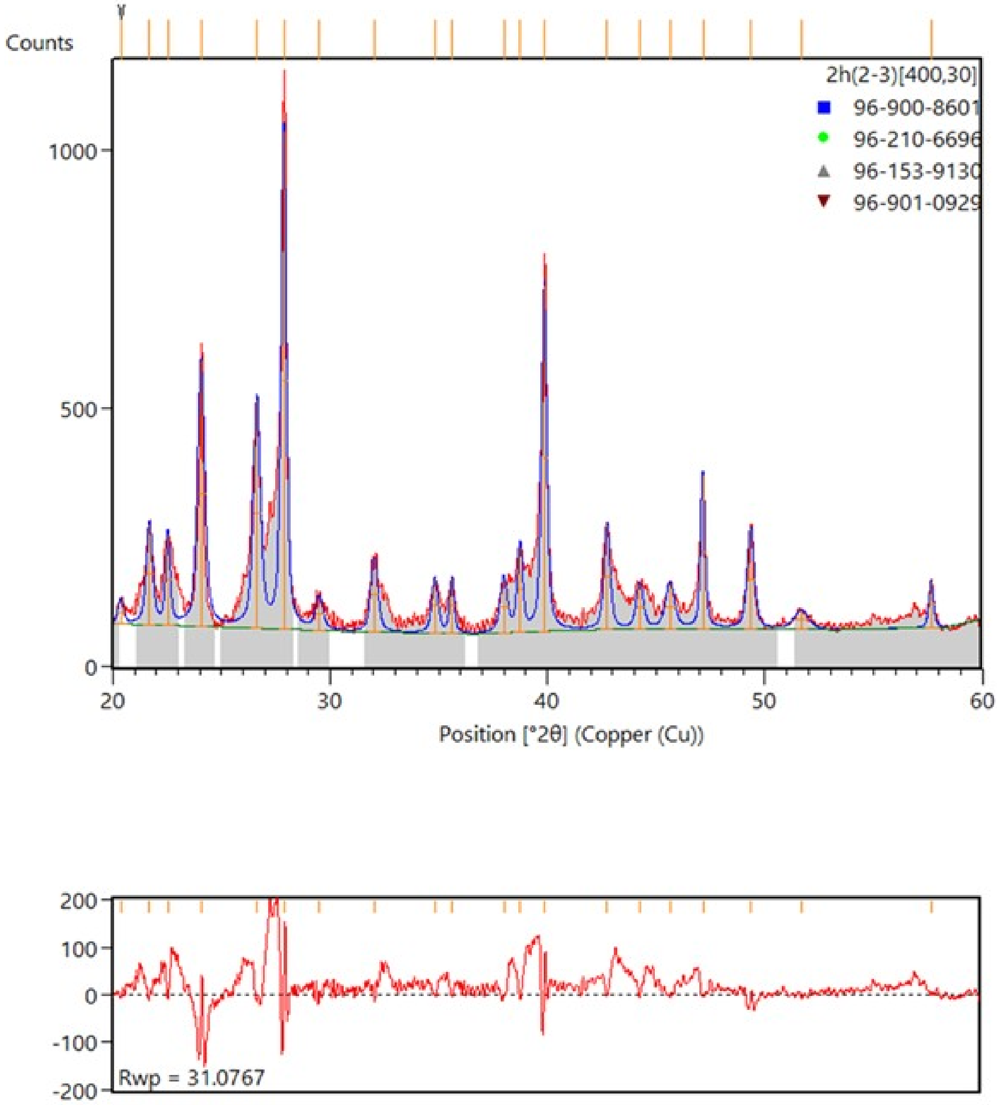

Figure S3: *Top: Experimental (red) and calculated (blue) XRD diffractograms of Sample 2: [400 °C, 30 mins] (2:3 $Ba(OH)_2$: S ratio, 2h milling). Bottom: Refinement residual plot.*

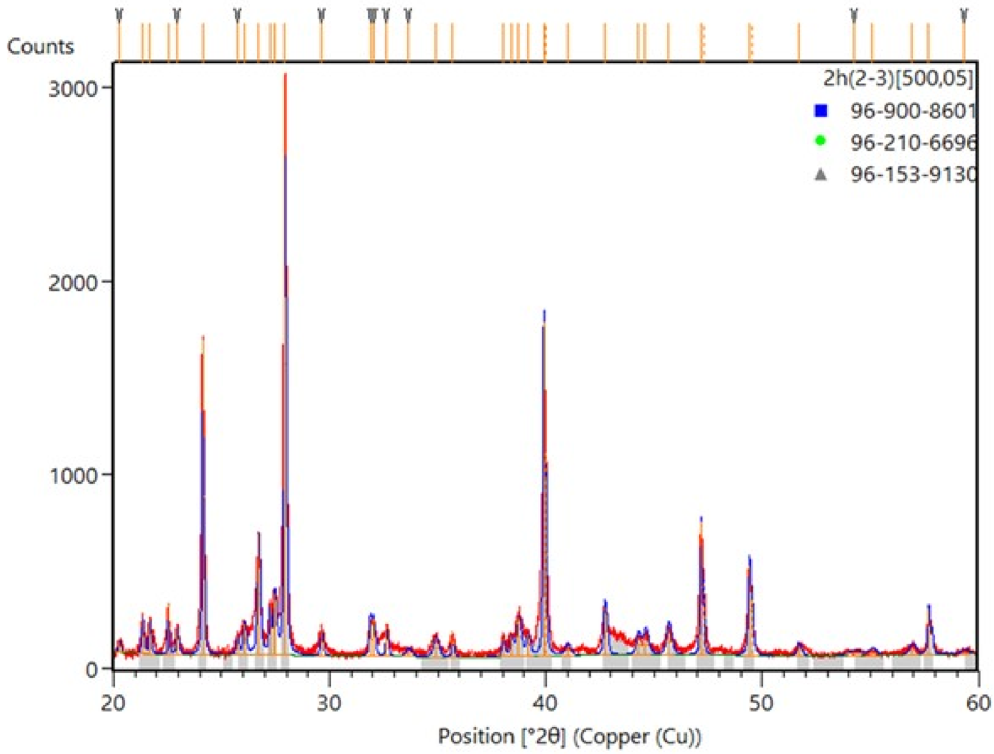

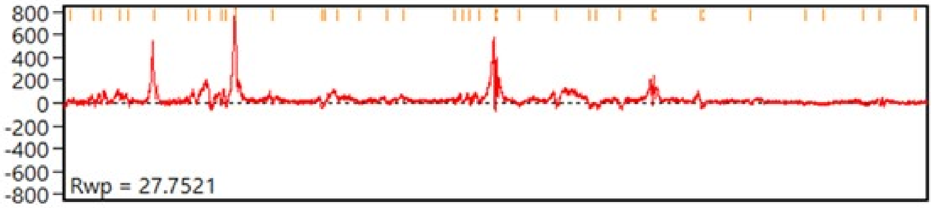

Figure S4: *Top: Experimental (red) and calculated (blue) XRD diffractograms of Sample 3: [500 °C, 05 mins] (2:3* $Ba(OH)_2$ *: S ratio, 2h milling). Bottom: Refinement residual plot.*

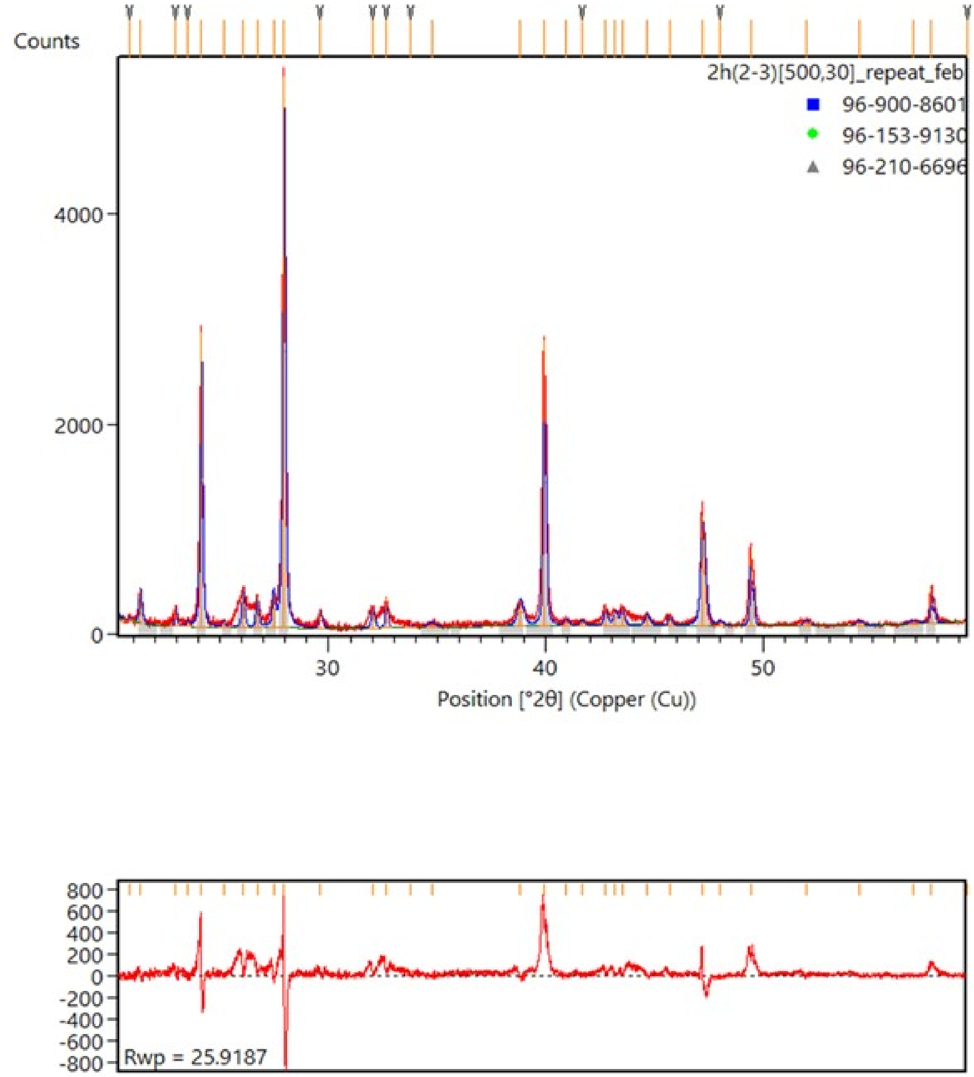

Figure S5: *Top: Experimental (red) and calculated (blue) XRD diffractograms of Sample 4: [500 °C, 30 mins] (2:3 Ba(OH)$_2$: S ratio, 2h milling). Bottom: Refinement residual plot.*

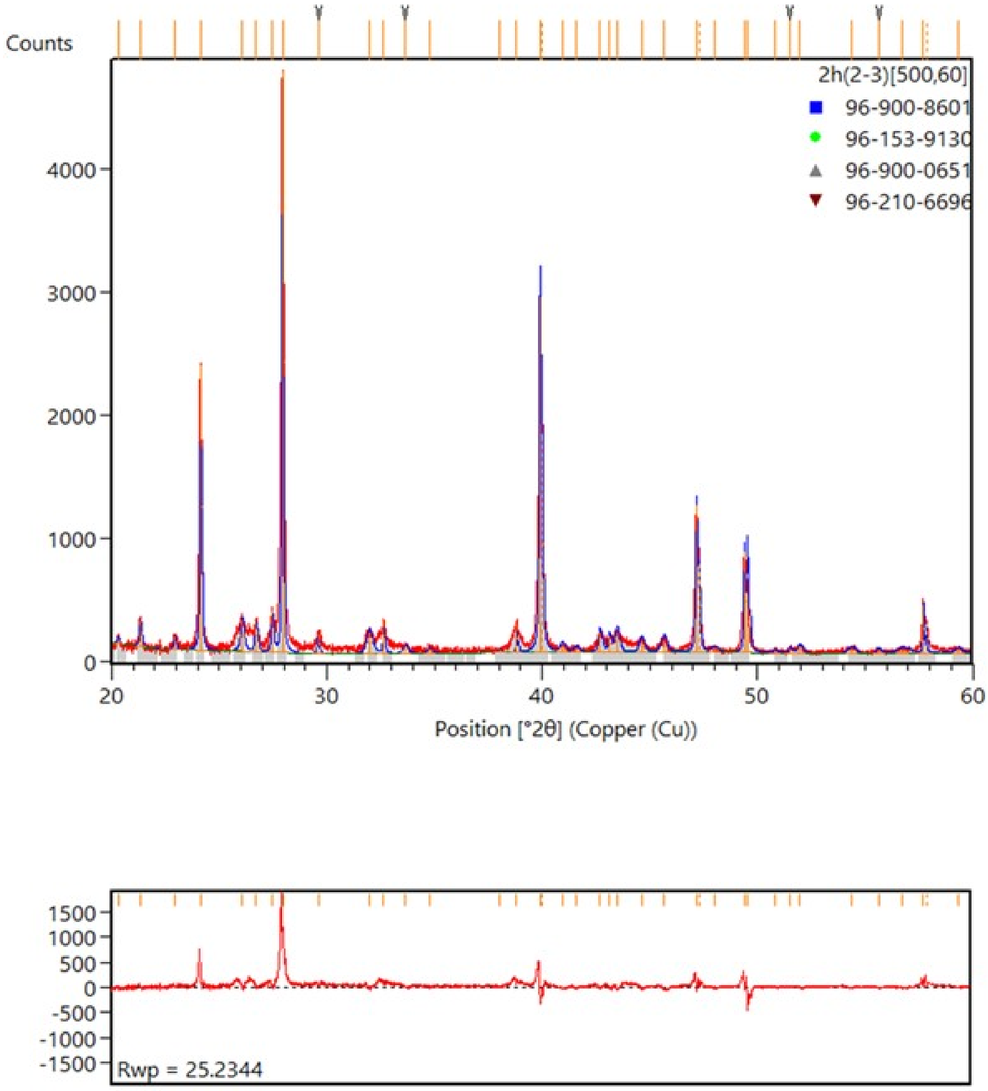

Figure S6: *Top: Experimental (red) and calculated (blue) XRD diffractograms of Sample 5: [500 °C, 60 mins] (2:3 Ba(OH)$_2$: S ratio, 2h milling). Bottom: Refinement residual plot.*

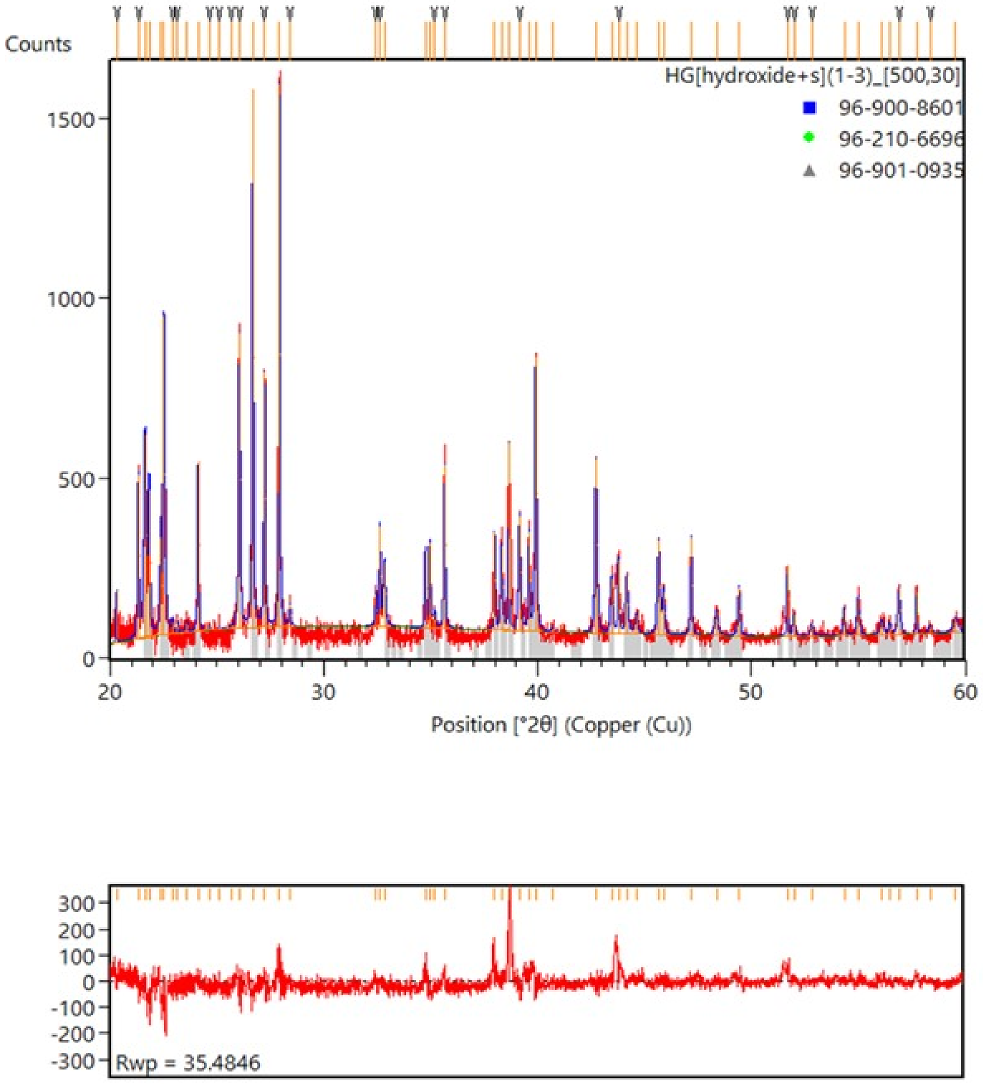

Figure S7*: Top: Experimental (red) and calculated (blue) XRD diffractograms of Sample 6: [500 °C, 30 mins] (1:3 $Ba(OH)_2$: S ratio, 0h milling). Bottom: Refinement residual plot.*

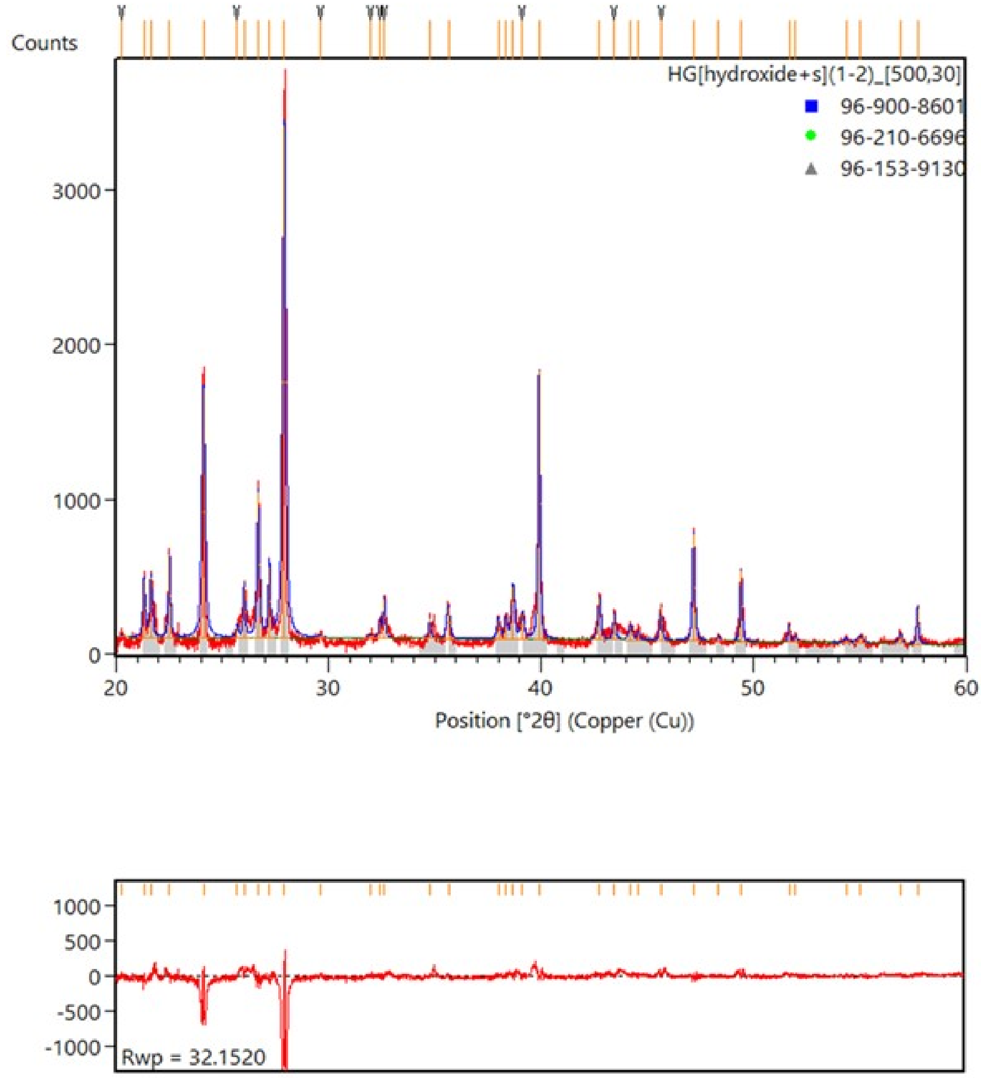

Figure S8: *Top: Experimental (red) and calculated (blue) XRD diffractograms of Sample 7: [500 °C, 30 mins] (1:2* $Ba(OH)_2$ *: S ratio, 0h milling). Bottom: Refinement residual plot.*

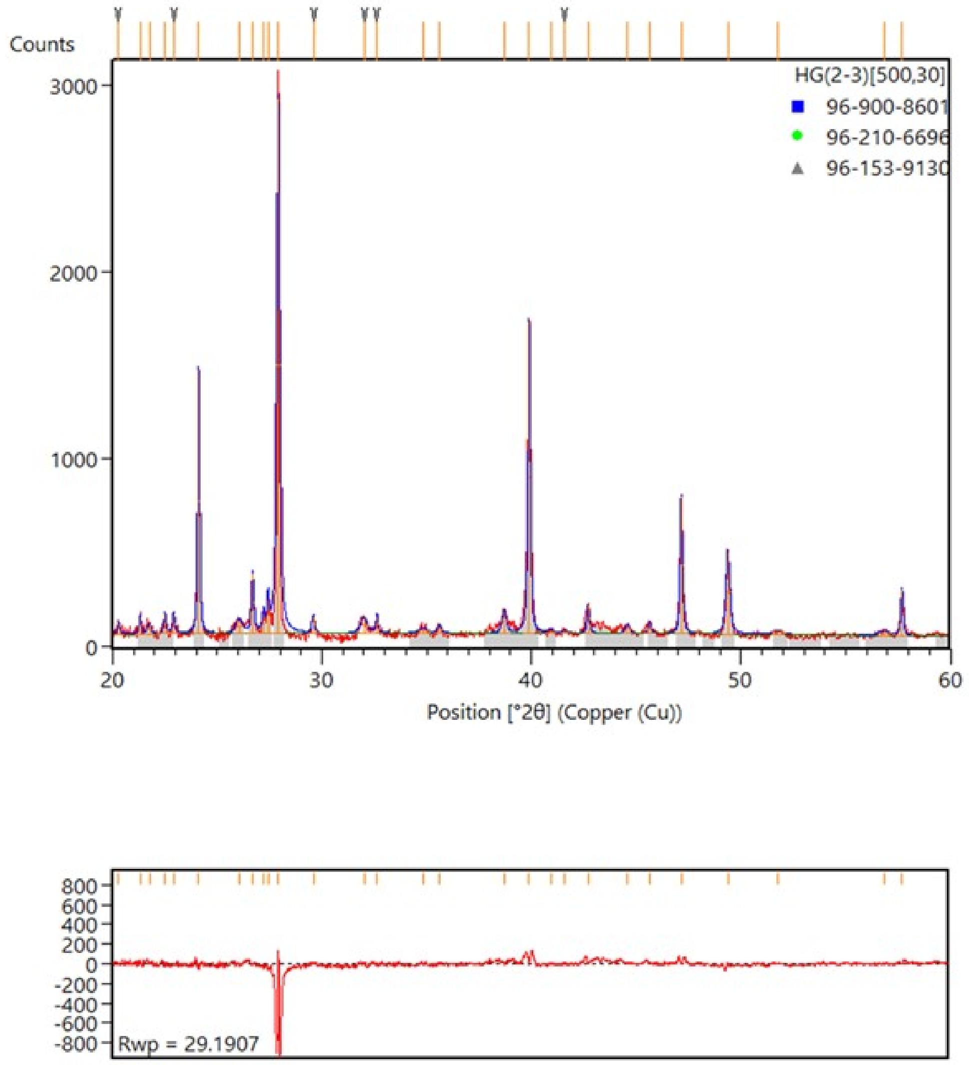

Figure S9: *Top: Experimental (red) and calculated (blue) XRD diffractograms of Sample 8: [500 °C, 30 mins] (2:3* $Ba(OH)_2$ *: S ratio, 0h milling). Bottom: Refinement residual plot.*

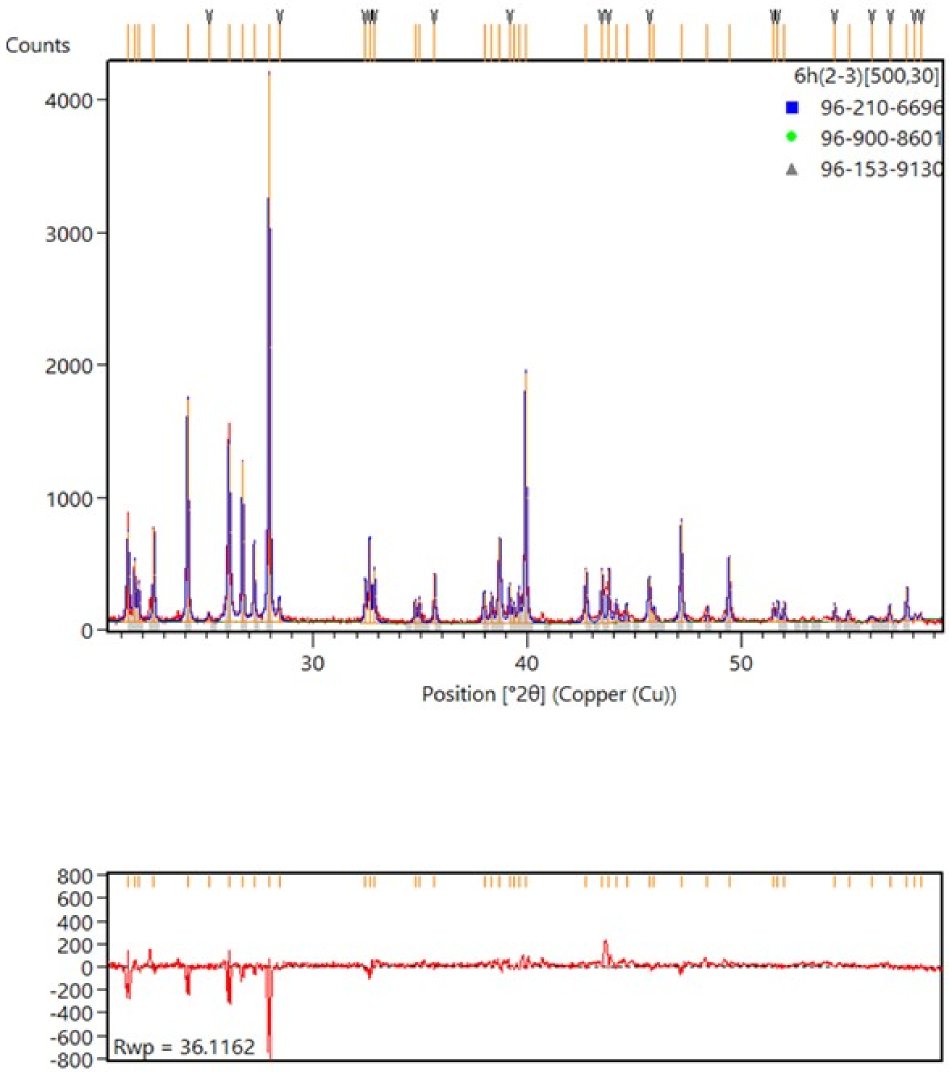

Figure S10: *Top: Experimental (red) and calculated (blue) XRD diffractograms of Sample 9: [500 °C, 30 mins] (2:3 $Ba(OH)_2$: S ratio, 6h milling). Bottom: Refinement residual plot.*

REFERENCE DIFFRACTOGRAMS

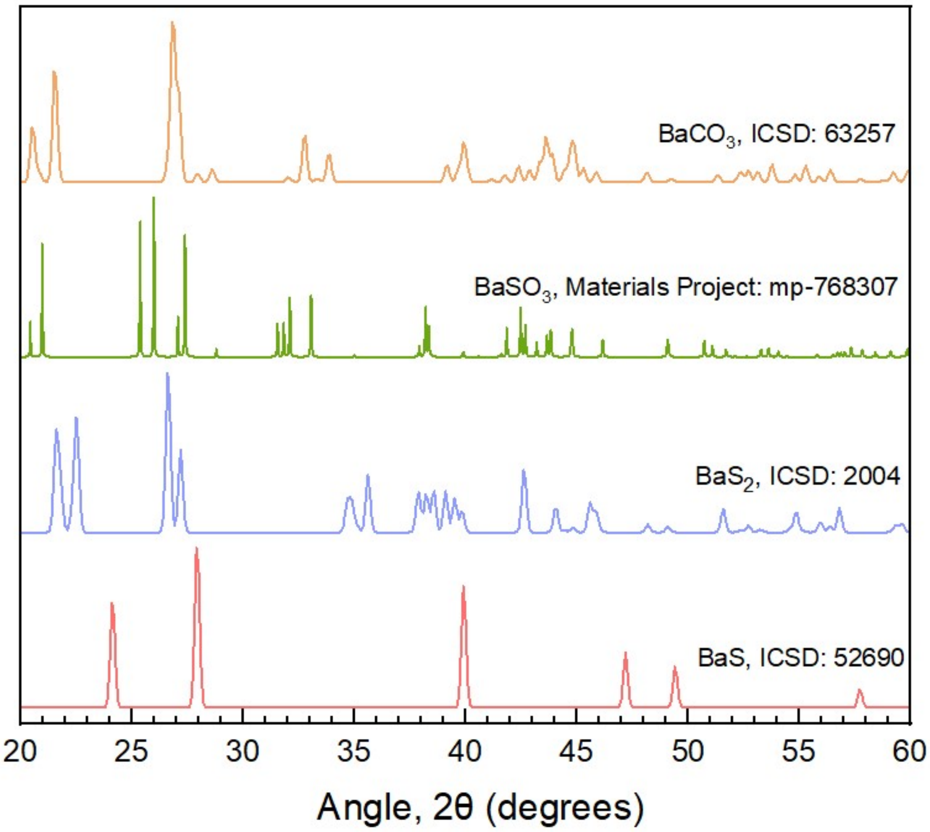

Figure S11: *XRD reference diffractograms of relevant phases: BaS (F m -3 m), BaS_2 (C 1 2/c 1) $BaSO_3$ (P2 1/m) and $BaCO_3$ (P 1 21/m 1) taken from ICSD and Materials Project.*

FTIR

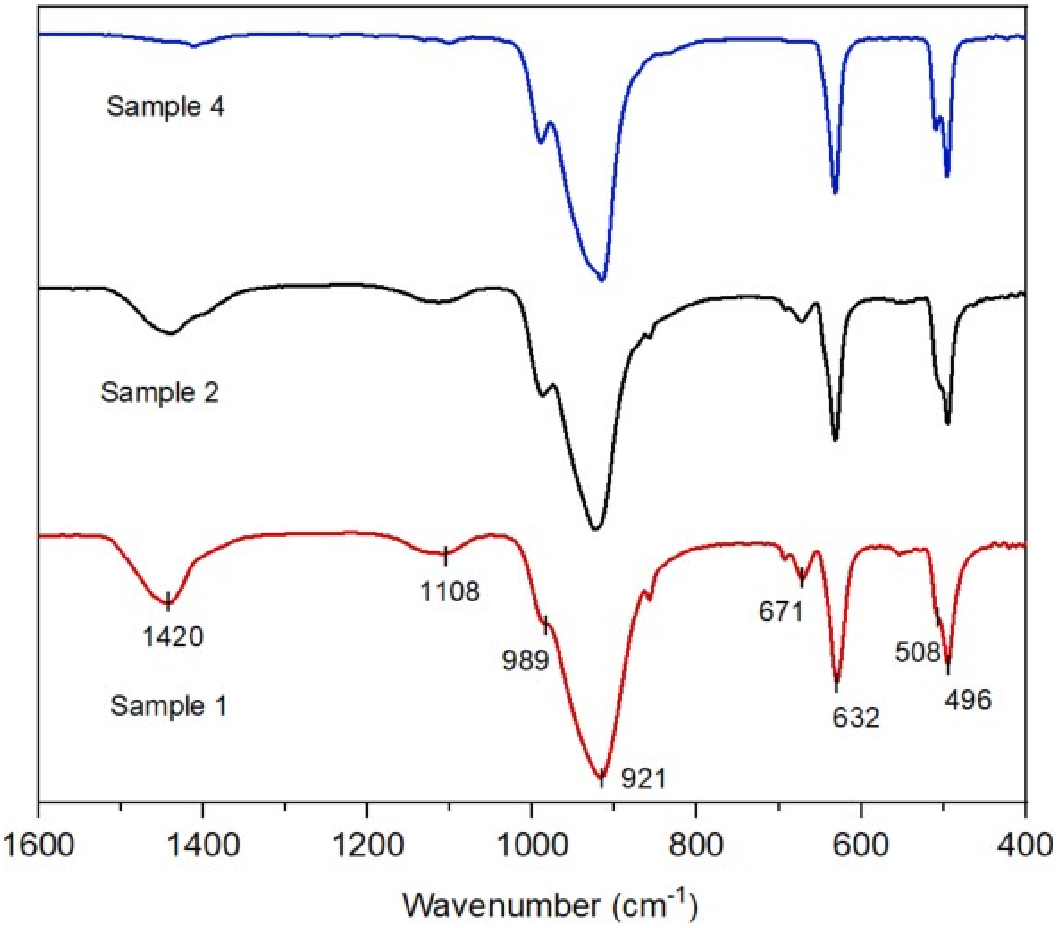

Figure S12: *FTIR spectra of temperature series. Samples 1 (300 ˚C), 2 (400 ˚C), and 4 (500 ˚C).*

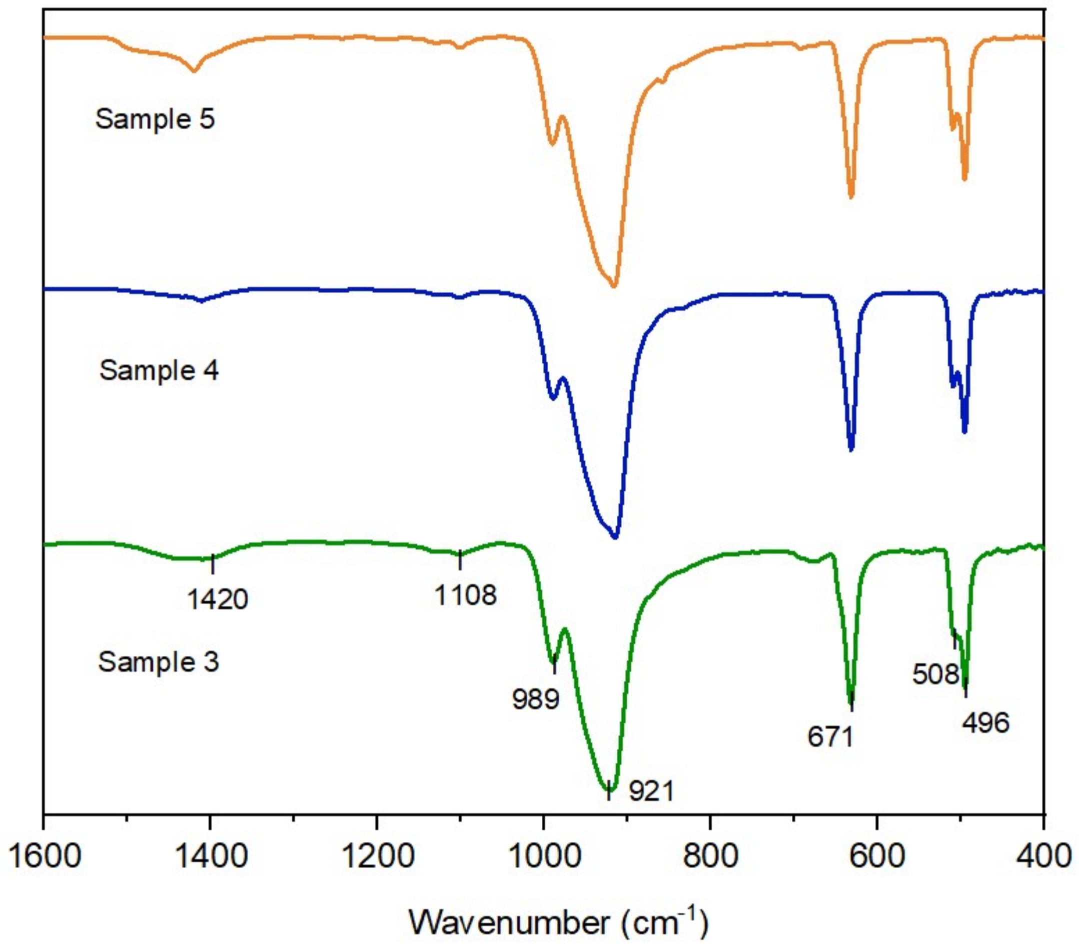

Figure S13: *FTIR spectra of time series. Sample 3 (5-minute dwell), 4 (30-minute dwell), and 5 (60-minute dwell).*

RAMAN ANALYSIS

Table S3: *Relative intensity ratios of Raman peak responding to BaS_2*

Sample ID	Intensity ratio of ~450cm^{-1} peak to ~200cm^{-1} peak	Quantified BaS_2 content
2	12.5	33%
3	11.4	19%
4	9.68	4%
5	8.55	3%

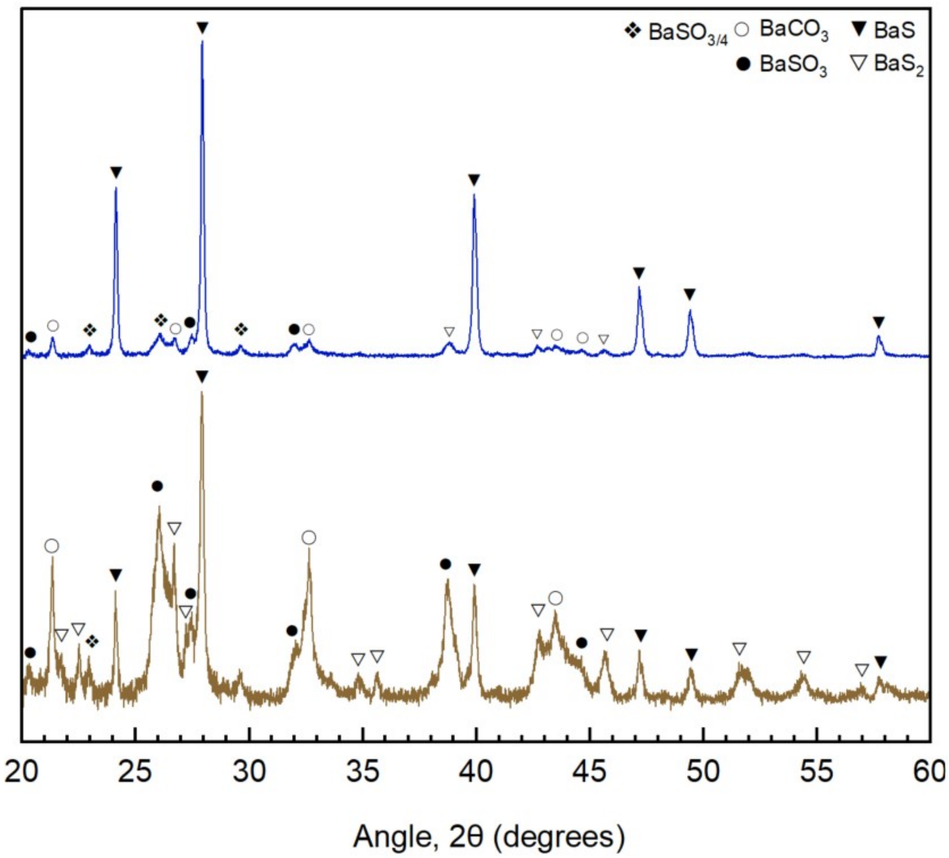

Figure S14: *Diffractograms of identical powder mixture annealed at 500 °C for 30 mins: as is described in the methodology (blue), and with the vial lid screwed on tightly, minimising gas flow (brown).*

RESIDUAL GAS ANALYSIS

For the Inductively Coupled Plasma Optical Emission Spectroscopy (ICP-OES) using the Gencoa Optix system the following emission wavelengths were used for each of the species (Table S4).

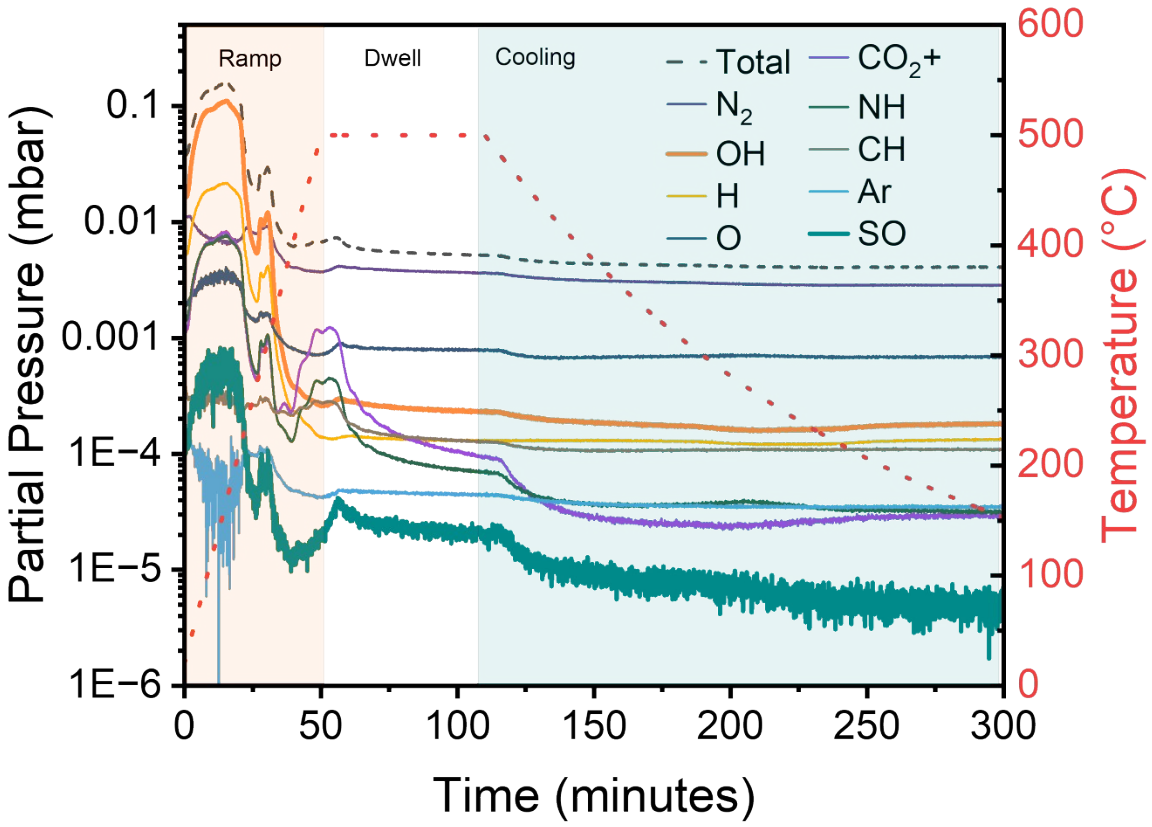

Figure S15: *Residual gas analysis (RGA) of the exhaust from the furnace tube during the temperature, dwell and part of the cool down period of the reaction for Sample 5. The red dotted line represents the temperature as a function of time. These temperatures have been estimated from the linear temperature increase during the 50-minute ramp period and the measured temperature of 500 °C for the dwell period. For the cooling period an exponential decrease in temperature via natural cooling was assumed.*

Table S4: *The emission wavelengths used to monitor each species during the ICP-OES measurement. All values used were provided by the manufacturer apart from 269.9 nm[a] SO which was obtained from work by E. V. Martin.[1]*

Species	Emission Wavelength (nm)
N_2+	391.3
O	777.7
H	656.6
OH	309.6
CO	561.1
CO_2+	289.6
Ar	750.4
N_2	336.9
CH	386.9
F	685.6

He	587.5
Ne	693.0
Cl	837.7
NH	325.0
C_2	516.2
SO	269.9[a]

REFERENCES

1. E. V. Martin, *Phys. Rev.,* 1932, *41,* 167
